# Biological effects of intraoperative radiation therapy: histopathological changes and immunomodulation in breast cancer patients

**DOI:** 10.3389/fimmu.2024.1373497

**Published:** 2024-04-24

**Authors:** Javier I. J. Orozco, Betsy J. Valdez, Chikako Matsuba, Michael P. Simanonok, Miquel Ensenyat-Mendez, Judi Anne B. Ramiscal, Matthew P. Salomon, Yuki Takasumi, Janie G. Grumley

**Affiliations:** ^1^ Saint John’s Cancer Institute, Providence Saint John’s Health Center, Santa Monica, CA, United States; ^2^ Department of Medicine, Keck School of Medicine, University of Southern California, Los Angeles, CA, United States; ^3^ Center for Cardiovascular Analytics, Research and Data Science, Providence Research Network, Portland, OR, United States; ^4^ Cancer Epigenetics Laboratory at the Cancer Cell Biology Group, Institut d’Investigació Sanitària Illes Balears (IdISBa), Palma, Spain; ^5^ Department of Surgical Oncology, Arrowhead Regional Medical Center & California University of Science and Medicine, Colton, CA, United States; ^6^ Department of Pathology, Providence Saint John’s Health Center, Santa Monica, CA, United States

**Keywords:** breast neoplasms, intraoperative radiation therapy-IORT, squamous metaplasia, immune response, tumor microenvironment

## Abstract

**Introduction:**

Intraoperative radiation therapy (IORT) delivers a single accelerated radiation dose to the breast tumor bed during breast-conserving surgery (BCS). The synergistic biologic effects of simultaneous surgery and radiation remain unclear. This study explores the cellular and molecular changes induced by IORT in the tumor microenvironment and its impact on the immune response modulation.

**Methods:**

Patients with hormone receptor (HR)-positive/HER2-negative, ductal carcinoma *in situ* (DCIS), or early-stage invasive breast carcinoma undergoing BCS with margin re-excision were included. Histopathological evaluation and RNA-sequencing in the re-excision tissue were compared between patients with IORT (n=11) vs. non-IORT (n=11).

**Results:**

Squamous metaplasia with atypia was exclusively identified in IORT specimens (63.6%, *p*=0.004), mimicking DCIS. We then identified 1,662 differentially expressed genes (875 upregulated and 787 downregulated) between IORT and non-IORT samples. Gene ontology analyses showed that IORT was associated with the enrichment of several immune response pathways, such as inflammatory response, granulocyte activation, and T-cell activation (*p*<0.001). When only considering normal tissue from both cohorts, IORT was associated with intrinsic apoptotic signaling, response to gamma radiation, and positive regulation of programmed cell death (*p*<0.001). Using the xCell algorithm, we inferred a higher abundance of γδ T-cells, dendritic cells, and monocytes in the IORT samples.

**Conclusion:**

IORT induces histological changes, including squamous metaplasia with atypia, and elicits molecular alterations associated with immune response and intrinsic apoptotic pathways. The increased abundance of immune-related components in breast tissue exposed to IORT suggests a potential shift towards active immunogenicity, particularly immune-desert tumors like HR-positive/HER2-negative breast cancer.

## Introduction

The standard treatment for patients with early-stage breast cancer includes breast-conserving surgery (BCS) followed by whole or partial breast radiotherapy. The benefit of radiotherapy relies upon reducing in-breast recurrences that predominantly occur in the post-excision lumpectomy cavity (“tumor bed”) (1). This has led to the notion that radiation directed only to the surgical tumor bed and the immediate surrounding area, namely the tumor microenvironment (TME), might be enough to mitigate the local recurrence. Intraoperative radiation therapy (IORT), which delivers a single dose of radiation directly to the tumor bed during the surgery immediately after removing the tumor, has emerged as an alternative to external beam radiation therapy for appropriately selected patients (2). The TARGIT-A study is a large randomized clinical trial comparing clinical outcomes of IORT vs. whole breast radiation therapy (WBRT) in patients with early-stage breast cancer. When performed at the time of BCS, IORT showed equivalent local recurrence-free survival, distant disease-free survival, breast cancer-specific mortality, and overall survival than WBRT, and reduced the burden of care (3, 4).

After radiation therapy, histologic changes in normal breast tissue involve epithelial elements, stroma, and vessels. Earlier reports have consistently described the presence of atypical epithelial cells in the terminal ductal lobular unit following WBRT (5–8). Additionally, squamous metaplasia—the transformation of glandular into a stratified squamous epithelium— with atypia has been reported after breast-conserving surgery and radiotherapy (8, 9). These benign findings can introduce challenges in pathology interpretation since they may mimic *in situ* carcinoma. Notably, these studies were based on biopsies obtained several months or years after the radiotherapy. However, the acute radiation-induced changes after IORT have not been fully explored, and the biological impact of these findings remains unknown.

IORT has sparked a rapid interest in the immediate biological effects of radiation therapy on the tumor bed and tumor microenvironment. Early *in vitro* studies mainly focused on the influence of surgical wound fluid (seroma) on breast cancer cell line models (10–12). They showed that wound surgical fluid from IORT decreased breast cancer cell growth and impaired cancer cell migration and invasiveness in comparison to seroma from non-IORT patients (10, 11). However, the synergistic effects of simultaneous surgery and radiation therapy under *in vivo* conditions on the tumor bed have been scarcely investigated.

In addition to the outright lethal effects on cancer cells, radiotherapy can also modulate the TME by either stimulating or suppressing the antitumor immune response (13, 14). The overall immunological impact of radiotherapy likely depends on many factors, including the tumor type and the modality and dose of radiotherapy (15, 16). These mechanisms have not been explored in IORT, and it is unclear if a localized single high dose of radiotherapy can also contribute to immune activation or suppression.

In this study, we explored the cellular and molecular changes induced by IORT in the TME and its impact on immune modulation. To accomplish this, we examined breast tissue specimens from patients undergoing re-excision for inadequate margins after BCS. We then compared histopathological and gene expression differences between patients undergoing IORT and those without IORT.

## Methods

### Patient selection

Patients with breast cancer undergoing breast-conserving surgery requiring a subsequent margin re-excision at a single institution (Providence Saint John’s Health Center) were selected. We included women with histologically confirmed diagnoses of ductal carcinoma *in situ* (DCIS) or invasive early-stage breast cancer (T1-T2, N0), without neoadjuvant systemic therapy, and undergoing BCS. Two cohorts were created according to the reception of IORT during the primary breast surgery into IORT vs. no-IORT (control group). IORT consisted of the delivery of 20 Gy immediately after the removal of the tumor through a brachytherapy balloon surface using the Xoft Axxent eBx^®^ System.

All clinical-demographic data and patient-derived samples were collected under research protocols approved by the Institutional Review Board of Providence Health and Services (Protocol #: STUDY2019000543). The experiments were performed according to the World Medical Association Declaration of Helsinki and the National Institutes of Health Belmont Report. Tissues were de-identified and coded according to the Health Insurance Portability and Accountability Act recommendations to ensure patient confidentiality.

### Histopathological evaluation

Formalin-fixed paraffin-embedded (FFPE) tissues were obtained from the specimen containing the re-excision procedure. The specimens’ hematoxylin and eosin (H&E) clinical diagnostic slides were reviewed by a breast pathologist (Y.T.). The initial assessment aimed to identify epithelial, stromal, and vascular changes in the re-excision tissue samples. Squamous metaplasia (SM) consists of squamous differentiation of breast epithelial cells, characterized by cells with dense cytoplasm with intercellular bridges. Squamous metaplasia with atypia (SMwA) was defined by the presence of atypical epithelial cells. The distribution of SM and SMwA according to extension was defined as focal (<20% of the slide), multifocal (20-80% of the slide), and diffuse (>80% of the slide).

Additional immunohistochemistry was performed at the pathologist’s discretion to further categorize the histologic findings. Briefly, 4 µm FFPE tissue slides were stained using a Ventana BenchMark ULTRA automated slide stainer (Roche Diagnostics, Indianapolis, IN, USA). Antibodies used were anti-estrogen receptor (SP1, #790-4324, Ventana Medical Systems, Tucson, AZ, USA) and anti-p63 (4A4, #K10259, Ventana Medical Systems, Tucson, AZ, USA).

### Tissue processing, RNA purification, and RNA sequencing

After deparaffinization, 8-10 μm-thick serial tissue sections were micro-dissected using labeled 4-μm thick H&E slides as a template. RNA was purified from the breast re-excision specimens from regions containing normal breast tissue, SM, and SMwA using the Quick-RNA FFPE kit (# R1008, Zymo Research). RNA was measured using the Qubit RNA Kit on a Qubit 4 Fluorometer (ThermoFisher Scientific). RNA sequencing (RNA-seq): RNA samples with high quality (RIN ≥8.0) and high purity (A260/280 >1.8) were used to generate libraries using KAPA HyperPrep Kit with RiboErase (rRNA depletion kit) for FFPE tissues and sequenced on an Illumina NovaSeq 6000 using 100 bp paired-end reads to a depth of 30~40 million reads.

### Statistical and bioinformatics analyses

Clinicopathologic characteristics were summarized by median with interquartile range (IQR) for continuous variables and frequency with percentage for categorical data. Comparisons between cohorts were performed using Fisher’s exact test for categorical variables. Raw RNA-seq reads were checked for quality using FastQC, filtered for adapters using Trimmomatic (17), and mapped to the human genome and annotation references GRCh38 using the STAR aligner (v.2.7.2b). Using gene-level read counts, hierarchical clustering with heatmaps, principal component analysis (PCA), differentially expressed genes (DEGs) analysis, and gene enrichment pathway analysis were generated on the R packages wrapper iDEP ver.96 (18). Differentially expressed genes (DEGs) were identified using the *DESeq2 (* 19). Genes with an absolute log-transformed fold change (Log_2_FC) ≥2 and an FDR<0.1 were considered significantly differentially expressed. Pathway analysis on the gene ontology (GO) biological process gene set was performed using the DEGs with ShinyGO (v0.77) (20). Additionally, gene pathway enrichment analysis was performed by applying the Generally Applicable Gene-set Enrichment (GAGE) method (21) using gene fold-change values independently of the selected DEGs. Transcripts per million (TPM) counts were generated using the Kallisto program (v0.46.00). From the TPM data, we determined the abundance of immune and stromal cell types in the breast tissue using the TIMER2.0 (22) and xCELL (23) algorithms for cell type enrichment analysis. The xCell algorithm deconvolutes the cellular composition into immune and stromal cells from tissue samples using gene expression data. Cell-type enrichment analyses also include the generation of the immune and stroma scores (23). Differences in the immune and stromal scores, as well as the enriched cell types between IORT and non-IORT samples, were compared using two-sample tests with Bonferroni *p*-value corrections for multiple comparisons. Statistical significance was set at *p*<0.05. All statistical analyses were performed using the R software, version 4.3.0 (R Core Team 2023).

## Results

### Patients and clinicopathologic characteristics

We selected a cohort of twenty-two women who underwent breast-conserving surgery followed by margin re-excision from October 2018 to March 2020. Of those, 11 patients (50%) were delivered IORT at the time of the primary breast surgery, and 11 patients (50%) did not receive IORT. The median age at diagnosis was 66 years (IQR= 61.3 – 71.8). The median time from the primary breast surgery to the re-excision procedure was 15 days (IQR= 13 – 20.8). Most tumors were invasive ductal carcinoma (N= 18, 81.8%), pathological stage I (N= 9, 40.9%), grade I (N= 10, 45.5%), and estrogen-receptor (ER) positive (N= 21, 95.4%, **Supplementary Table 1**). All cases in the IORT cohort were ER-positive/HER2-negative.

### Histopathological changes after IORT

**Figure 1:**
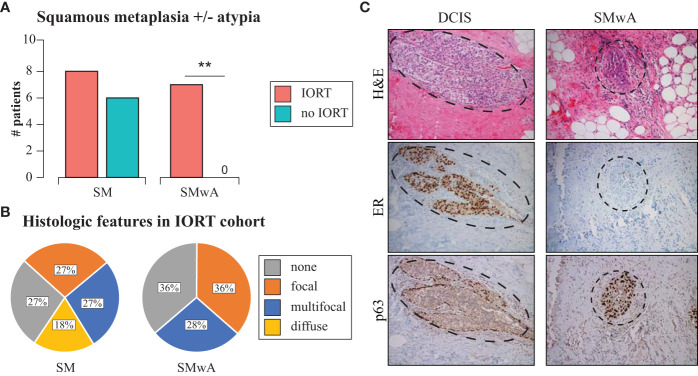
Histopathological changes after IORT. **(A)** Barplots of SM and SMwA in patients with and without IORT. Fisher’s exact test **p=0.004. **(B)** Distribution of SM and SMwA according to extension as focal (<20% of the slide), multifocal (20-80% of the slide), and diffuse (>80% of the slide). **(C)** Representative staining of DCIS (left panel) and SMwA (right panel) with differential expression of immunohistochemistry markers (ER and p63). IORT, intraoperative radiation therapy; SM, squamous metaplasia; SMwA, squamous metaplasia with atypia; DCIS, ductal carcinoma in situ; H&E, hematoxylin and eosin; ER, estrogen receptor.

When assessing histologic changes from the clinical diagnostic re-excision tissue slides, there was a higher, but not statistically significant, proportion of squamous metaplasia (SM) in the IORT cohort (72.7%) vs. the non-IORT cohort (54.5%, *p*=0.66). Remarkably, the presence of squamous metaplasia *with atypia* (SMwA) was exclusively observed in IORT specimens (63.6%, *p*=0.004, **Figure 1A**), with various degrees of extension (**Figure 1B**). Of note, SMwA mimicked ductal carcinoma *in situ* (DCIS) on histology, requiring complementary stains such as ER and p63 to confirm the diagnosis (**Figure 1C**). Neither SM nor SMwA were found in the specimens from the initial breast surgery.

### Transcriptomic changes after IORT

**Figure 2:**
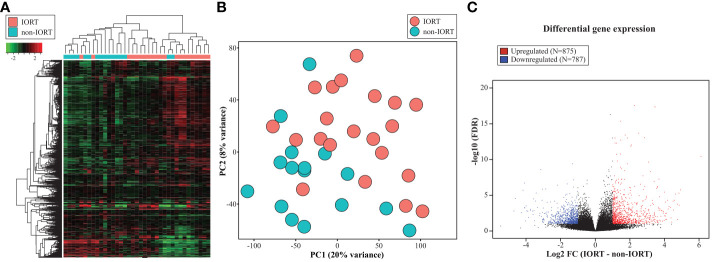
Transcriptomic changes after IORT. Heatmap representing the hierarchical clustering **(A)** and principal component analysis (PCA) **(B)** using the 1,000 most variable genes. **(C)** Volcano plot showing the differentially expressed genes (DEG) between IORT and non-IORT samples (FDR<0.1).

**Figure 3:**
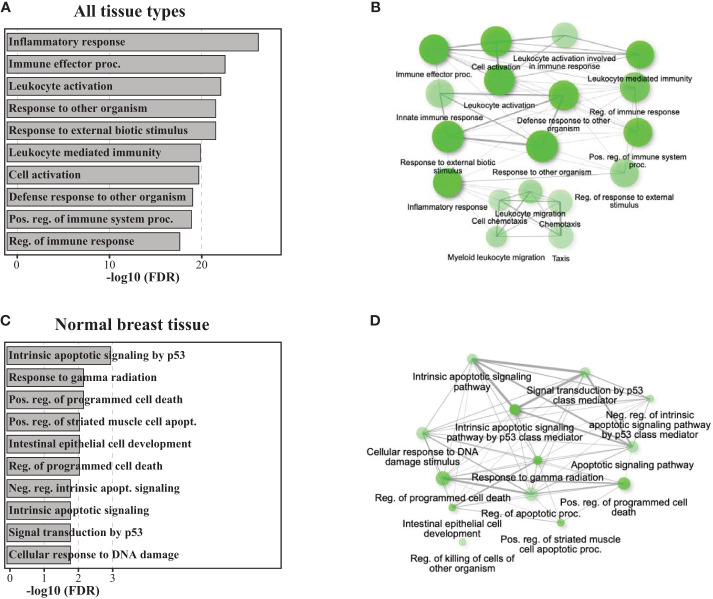
Pathway analyses of gene ontology biological processes. Barplots and network plots showing upregulated gene enrichment pathways using the DEGs for gene ontology (GO) biological process including all tissue types (IORT=21, non-IORT=16) in panels **(A, B)**, and only normal breast tissue (IORT=6, non-IORT=11) in panels **(C, D)**, respectively. Network plots only show significantly upregulated pathways (in green) in IORT samples. Two pathways (nodes) are connected if they share 20% or more genes. Darker nodes indicate the lower adjusted p-value, a larger size of the circle indicates a larger number of genes found in the pathway, and thicker edges represent more overlapped genes.

When applying RNA sequencing in the re-excision tissue, using the 1,000 most variable genes, we identified a significant separation of samples according to the exposure to radiation therapy (**Figures 2A, B**). When including all tissue samples (i.e., SM, SMwA, and normal tissue), we found 1,662 differentially expressed genes (875 upregulated and 787 downregulated) between IORT and non-IORT samples (**Figure 2C**). The gene ontology analyses using those DEGs showed that IORT samples were significantly associated with the enrichment of several immune pathways, such as inflammatory response, immune effector process, leukocyte activation, and response to external stimulus (*p*<0.001; **Figures 3A, B**; **Supplementary Table 2**). Similarly, when using the gene fold change values, independently of the DEGs, the most significant enriched pathways in IORT samples were granulocyte activation, adaptive immune response, and T-cell activation (**Supplementary Table 3**). Notably, when only considering normal tissue from both cohorts, IORT was associated with intrinsic apoptotic signaling by the p53 mediator, response to gamma radiation, and positive regulation of programmed cell death (*p*<0.001; **Figures 3C, D**).

### Cell type enrichment in the tumor microenvironment

**Figure 4:**
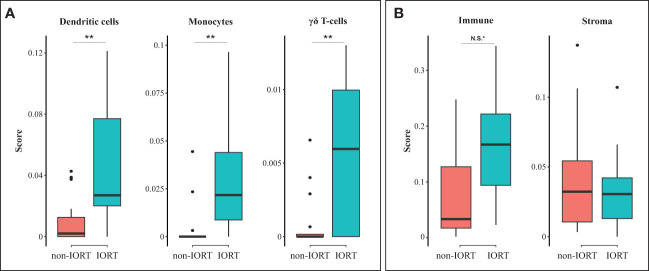
Cell type enrichment in the tumor microenvironment. Boxplots showing differences between non-IORT and IORT cohorts in immune cell enrichment **(A)** and immune and stroma scores **(B)**, as determined by the xCell digital portrayal algorithm from TIMER2.0. T-test with Bonferroni corrected values; **p < 0.05, *N.S., non-significant (*p* = 0.07).

When evaluating the cell type scores, using the xCell digital portrayal algorithm, we identified a higher abundance of gamma delta T-cells, dendritic cells, and monocytes in the IORT cohort compared to non-IORT samples (**Figure 4A**). This may imply a role of IORT in the modulation of adaptive and innate immunity. Other immune and stromal cells were not statistically significantly different between IORT and non-IORT samples (**Supplementary Table 4**). Additionally, we found suggestive evidence of higher immune-related components (“immune score”) in breast tissue samples exposed to IORT in comparison to non-IORT samples (0.17 vs 0.07, adjusted *p*=0.07; **Figure 4B**). Interestingly, there was no difference in the stromal score (0.03 vs 0.04, adjusted *p*=1; **Figure 4B**), which may reflect the influence of the surgical procedure in both cohorts in the wound-healing process.

## Discussion

In this study, we explored the intricate interplay between intraoperative radiation therapy (IORT) and the immune landscape of the tumor microenvironment (TME) in patients with hormone receptor-positive/HER2-negative early-stage invasive breast carcinoma, a traditionally considered immune-desert or “cold” tumor (24). The investigation focused on tissue specimens obtained from patients undergoing re-excision for inadequate margins after breast-conserving surgery (BCS). We explored the cellular and molecular alterations induced by IORT, shedding light on its potential impact on immune modulation.

Early studies conducted on core biopsy or mastectomy specimens after breast-conserving surgery and radiation therapy have revealed several radiation-induced histological changes in the breast tissue (5–8). Atypical epithelial cells were observed in 84% to 100% of irradiated breast tissue (5, 8). These findings seem specific to radiation since no epithelial atypia was observed in non-radiated breast tissue. Importantly, these changes were observed up to more than six years after radiation, suggesting the absence of regression of these lesions (8). Overall, these initial descriptions came from studies performed several months or years after the primary local therapy from breast tissue samples obtained during the 1980s and 1990s with radiation techniques that differ from current modern ones.

The immediate histologic changes following breast IORT have been scarcely described in the literature. Ginter et al. reported the presence of squamous metaplasia in ten of twelve cases (83%) collected within six months of IORT (25). The presence of SMwA was identified in five cases (33%). Of note, this study lacked a non-IORT cohort as a control comparator. Our study showed no differences in the presence of squamous metaplasia in patients who have undergone IORT compared to those who have not, which may reflect the effect of surgery on both cohorts. Noteworthy is that squamous metaplasia with atypia (SMwA) was exclusively found in tissue specimens of 64% of patients receiving IORT. Like previous reports describing the presence of epithelial atypia after radiation therapy (5–9), SMwA resembled ductal carcinoma *in situ* (DCIS), warranting additional staining, such as ER and p63, for accurate diagnosis. Our findings align with previous reports on the effects of external beam radiation, emphasizing the need for comprehensive understanding and recognition of acute and chronic changes induced by radiation therapy. In considering atypical changes in the breast tissue of patients who have had breast cancer and in whom residual disease is suspected, it is crucial to distinguish the epithelial changes due to radiation from those of cancer.

The impact of the molecular and cellular changes induced by IORT on the breast has been mainly focused on the influence of surgical wound fluid (seroma) on breast cancer cell lines. Belletti et al., evaluating 45 patients from the TARGIT-A trial, demonstrated that breast cancer cells incubated in seroma obtained from IORT-treated tumor beds decreased their proliferation, migration, and invasiveness capacity compared to seroma from non-IORT patients (10). Similarly, Kulcenty et al. found that IORT impaired the epithelial-mesenchymal transition (EMT) induced by wound fluids (11). These and other studies have also demonstrated a differential profile of cytokines and growth factors in the IORT-seroma, halting the tumor growth (10, 26, 27). Contrarily, Veldwijk et al. found no significant differences in proliferation and clonogenic growth capacity of breast cancer cell lines incubated in IORT vs. non-IORT seroma (28). Of note, these *in vitro* models may not fully represent the complexity of the *in vivo* tumor microenvironment, including interactions with the immune system and other surrounding tissues, and lack the clinical heterogeneity of breast cancer in female patients. In our study, to gain insights into the influence of IORT on the TME under *in vivo* conditions, we performed a transcriptomic analysis of the breast tissue obtained 15 days (median) after the IORT. These tissues represent a unique resource to explore differences in the TME via a combination of standard histopathological techniques and next-generation sequencing.

Radiotherapy is an efficient modulator of the immune response that may show antagonistic effects by facilitating or suppressing the anti-cancer immune response (13, 14). These opposed effects in the TME seem to be influenced by the radiation modality, field, dose, and fractionation schedule, among other factors (15, 16). As recently reviewed, conventionally fractionated radiotherapy can mediate an immunosuppressive effect by driving the recruitment and differentiation of immunosuppressive cells (e.g., M2-like tumor-associated macrophages, regulatory T cells, and exhausted T cells), hypoxia-driven resistance of cytotoxic T cells, and extratumoral immunosuppression by radiating tumor-draining lymph nodes and blood vessels included in the radiation field (15, 16). Conversely, focal radiotherapy delivered in a single or a few fractions has been associated with the upregulation of neoantigen-encoding genes, which enhances cancer cell antigenicity, induction of immunogenic cell death (ICD), and increased MHC class I molecules, which improves cancer cell recognition by CTLs (15, 16). In line with this notion, we found that a single dose of radiation limited to the tumor bed was associated with the enrichment of several immune response pathways, such as granulocyte and T-cell activation. Additionally, the xCell algorithm revealed a higher immune score, though not statistically significant, in breast tissues exposed to radiotherapy. Altogether, these findings reinforce the notion that IORT may induce a robust immune response within the TME.

At the cellular level, some initial investigations have evaluated the local and systemic effects of IORT in breast cancer. Linares-Galiana et al. assessed the changes in peripheral blood immune cell composition before and after IORT by flow cytometry in patients with early-stage ER-positive/HER2-negative breast cancer (29). They found that radiation therapy increased the presence of peripheral NK cells while no changes were detected in the immunosuppressive cells, such as regulatory T cells and myeloid-derived suppressor cells (29). In our study, which included a similar cohort of breast cancer patients, we inferred a significant enrichment of dendritic cells, essential for priming and maintaining the T cell response (30); gamma delta T-cells, which harbor antitumoral activity independent of the tumor mutational burden and the MHC class I- mediated antigen presentation (31); and monocytes, which may have dual antitumoral or protumor properties (32). Altogether, these observations suggest a potential shift towards active immunogenicity, involving innate and adaptive immunity, in traditionally “immune desert” tumors, such as HR-positive/HER2-negative breast cancer.

One of the pivotal mechanisms through which radiation therapy exerts its anti-cancer effects is the induction of apoptosis or programmed cell death (33). Ionizing radiation causes DNA damage within cancer cells, triggering a cascade of cellular responses, including activation of the tumor suppressor protein p53 (33). Notably, the response of the neighbor tissue, such as the normal breast tissues, to radiation exposure can further support the therapeutic effects of radiotherapy (34). In our study, when exclusively evaluating the normal breast tissue exposed to IORT, we observed an association with intrinsic apoptotic signaling by the p53 mediator, response to gamma radiation, and positive regulation of programmed cell death. Our findings corroborate previous *in vitro* and *in vivo* studies suggesting that IORT may induce apoptosis (12, 35). In a pilot study evaluating five patients, Shanai et al. performed a transcriptomic and proteomic analysis analyzing normal breast tissue before and immediately after IORT. They identified changes in several pathways linked to programmed cell death and cell cycle arrest after IORT (35). These findings highlight a potential link between IORT and programmed cell death, suggesting a nuanced interplay between radiation-induced changes and immune activation.

While this study sheds light on the cellular and molecular changes in breast tissue after IORT, several limitations should be acknowledged. Notably, all the patients included in our study had HR-positive/HER2-negative tumors, classically considered immune desert carcinomas. This is mainly due to the current indications of IORT, which are limited to patients with small tumors, cancer-free margins, and negative lymph nodes (2). Future studies should aim to capture the immunomodulatory effects in more immunogenic breast cancer subtypes, such as high-risk HR-positive/HER2-negative, HER2-positive, and triple-negative breast carcinomas. However, it will be challenging to enroll those patients since most of them lack current indications of IORT. While our findings provide valuable insights into the acute effects of IORT on the TME, further studies are warranted to elucidate the long-term implications and clinical outcomes associated with the observed immune modulation. Additionally, understanding the specific immune cell populations and their spatial location in relation to the tumor (i.e., within the tumor vs. surrounding the tumor) could pave the way for targeted immunotherapeutic approaches in IORT-treated breast cancer. Our study did not address this since most tissue samples included for margin re-excision did not contain residual cancer. The small sample size also limited our hypothesis-generating study and was not specifically designed to evaluate clinical outcomes in patients with breast cancer. Further studies correlating molecular changes with patient outcomes are warranted.

In conclusion, our study sheds light on the immediate effects of IORT in patients with HR-positive/HER2-negative early-stage invasive breast carcinoma, a traditionally considered immune-desert tumor. IORT induces histological changes, including squamous metaplasia with atypia, and elicits molecular alterations associated with intrinsic apoptotic signaling and immune response pathways. The increased abundance of immune-related components in breast tissue exposed to IORT suggests a potential shift toward active immunogenicity. This provides crucial insights into the immunomodulatory effects of IORT, offering a foundation for future investigations into tailored immunotherapies and combination approaches to optimize its therapeutic benefits in breast cancer treatment.

## Data availability statement

The RNA‐seq transcriptomic data (raw FASTQ and table counts) have been deposited to the Gene Expression Omnibus (GEO) repository under the accession number GSE253650.

## Ethics statement

The studies involving humans were approved by Institutional Review Board of Providence Health and Services. The studies were conducted in accordance with the local legislation and institutional requirements. The participants provided their written informed consent to participate in this study.

## Author contributions

JO: Writing – original draft, Writing – review & editing, Conceptualization, Data curation, Funding acquisition, Investigation, Methodology, Project administration, Resources, Supervision, Visualization. BV: Writing – original draft, Writing – review & editing, Investigation, Methodology, Resources. CM: Writing – original draft, Writing – review & editing, Data curation, Formal Analysis, Investigation, Software, Validation, Visualization. MSi: Writing – original draft, Writing – review & editing, Formal analysis, Investigation, Software, Validation, Visualization. ME-M: Writing – original draft, Writing – review & editing, Formal analysis, Software, Validation. JR: Writing – original draft, Writing – review & editing, Conceptualization, Data curation, Investigation, Methodology, Visualization. MSa: Writing – original draft, Writing – review & editing, Formal analysis, Supervision. YT: Writing – original draft, Writing – review & editing, Conceptualization, Data curation, Investigation, Resources. JG: Writing – original draft, Writing – review & editing, Conceptualization, Funding acquisition, Project administration, Supervision.
